# Anterior vertebral tethering for adolescent idiopathic scoliosis: our initial ten year clinical experience

**DOI:** 10.1007/s43390-024-00897-w

**Published:** 2024-05-26

**Authors:** John T. Braun, Sofia C. Federico, David M. Lawlor, Nikolaos J. Paschos, Daniel P. Croitoru, Brian E. Grottkau

**Affiliations:** 1 Massachusetts General Hospital, Harvard Medical School, 55 Fruit St., Yawkey 3E, Boston, MA 02114 USA; 2 Dartmouth Hitchcock Medical Center, Geisel School of Medicine at Dartmouth, Lebanon, NH USA

**Keywords:** Adolescent idiopathic scoliosis, Anterior vertebral tethering, Fusionless scoliosis surgery

## Abstract

**Background:**

Anterior vertebral tethering (AVT) is a minimally invasive alternative to fusion surgery for adolescent idiopathic scoliosis (AIS) that offers the potential for definitive scoliosis treatment with the possibility of preservation of the growth, motion, function and overall health of the spine. This study represents our first ten years using AVT to treat AIS.

**Methods:**

In this retrospective review we analyzed our first 74 AIS patients treated with AVT 2010–2020. Multiple Lenke curve types 33–70° were treated with skeletal maturity spanning Risser -1 to 5.

**Results:**

Of 74 consecutive AIS patients treated with AVT, 52 patients (47 female, 5 male) had sufficient 2-year follow-up for inclusion. Forty-six of these 52 patients (88%) with 65 curves (35T, 30TL/L) were satisfactorily treated with AVT demonstrating curve correction from 48.6° pre-op (range 33°–70°) at age 15.1 years (range 9.2–18.8) and skeletal maturity of Risser 2.8 (range -1 to 5) to 23.2° post-op (range 0°–54°) and 24.0° final (range 0°–49°) at 3.3 years follow-up (range 2–10 years). Curve corrections from pre-op to post-op and pre-op to final were both significant (p < 0.001). The 0.8° change from post-op to final was not significant but did represent good control of scoliosis correction over time. Thoracic kyphosis and lumbar lordosis were maintained in a normal range throughout while axial rotation demonstrated a slight trend toward improvement. Skeletal maturity of Risser 4 or greater was achieved in all but one patient. Four of the 52 patients (8%) required additional procedures for tether rupture (3 replacements) or overcorrection (1 removal) to achieve satisfactory treatment status after AVT. An additional 6 of the 52 patients (12%), however, were not satisfactorily treated with AVT, requiring fusion for overcorrection (2) or inadequate correction (4).

**Conclusions:**

In this study, AIS was satisfactorily treated with AVT in the majority of patients over a broad range of curve magnitudes, curve types, and skeletal maturity. Though late revision surgery for overcorrection, inadequate correction, or tether rupture was not uncommon, the complication of overcorrection was eliminated after our first ten patients by a refinement of indications.

**Level of Evidence:**

IV

## Introduction

Anterior vertebral tethering (AVT) has been proposed as a minimally invasive alternative to fusion surgery for adolescent idiopathic scoliosis (AIS) as it offers the potential for definitive scoliosis treatment with the possibility of preservation of growth, motion, and function of the spine. Additionally, and in contrast to a rigid, multi-level instrumented spinal fusion, flexible correction of spinal deformity with AVT may also preserve the overall health of the spine by reducing the future risk of adjacent segment degeneration requiring revision surgery [1–14]. Evidence in support of this novel procedure has steadily accumulated over the past two decades following an arc of translational research from bench to bedside [15–65].

Before we performed the first successful endoscopic AVT procedure for AIS in 2010 [43, 44], we had the benefit of insight and experience gained from more than a decade of our own basic science research and preclinical testing investigating a number of fusionless scoliosis surgery strategies. Our studies, as well as those of others, demonstrated the safety and efficacy of multiple fusionless techniques in creating, correcting, and controlling scoliosis in animal models [15–21]. Additionally, we had the benefit of reviewing a decade of related clinical information on vertebral stapling from Betz [39–41] and a case report on vertebral tethering from Crawford [42]. While Betz was able to demonstrate the relative safety and efficacy of vertebral stapling in AIS, their group was unable to demonstrate consistent correction or control of curves > 35°. And though Crawford was able to demonstrate substantial correction of a 40° curve after vertebral tethering in an eight-year-old male with juvenile idiopathic scoliosis (JIS), significant skeletal immaturity in this patient eventually led to overcorrection requiring tether removal. Two additional JIS patients were treated by this same group and, by report, also required tether removal for overcorrection (personal communication, Lenke, LG). Despite limitations, these clinical reports provided support for the concept that the abnormal and asymmetric spinal growth of idiopathic scoliosis could be harnessed and redirected with a convex tether to achieve correction, rather than progression, of deformity, according to Hueter-Volkmann [22–31].

Over the past decade, multiple clinical studies have demonstrated the relative safety and efficacy of AVT in the treatment of AIS curves averaging 37°–52° with good initial correction (44–58%) and subsequent control (8–24% additional correction with growth) of scoliosis over 1–5 years but with significant differences in rates of complications (12–48%) and additional procedures (12–41%) [43–65]. Though these early published series provide some insight into the challenges of AVT, the limitations of these studies are significant. Most of these early investigations have involved small, retrospective analyses that represent the initial learning curve of a particular center or group of centers without substantive critique of indications, refinement of techniques, or an evolution in care to improve outcomes and reduce complications over time. Further, these early published reports have primarily focused on a narrow segment of the AIS population. That is, patients with a single thoracic curve pattern (Lenke 1A) treated at the extreme of skeletal immaturity (often Risser -1). This almost exclusive targeting of single thoracic curves in patients on the cusp of an exponential increase in spinal growth velocity not only increased the likelihood of clinically significant overcorrection, progression after tether rupture, and adding-on, but also the potential for deformity progression in a distal, unrecognized, or neglected, thoracolumbar or lumbar curve. To our knowledge, we are the only group that has consistently used AVT to treat multiple single and double AIS curve types spanning the Lenke classification across the full range of the Risser skeletal maturity assessment for more than 10 years.

The purpose of this study was to analyze all AIS patients treated with AVT by a single lead surgeon over a full decade. Our hypotheses were: 1. AVT would satisfactorily treat AIS without fusion during the study period in the majority of patients by providing significant initial correction and subsequent control of progressive curves in the 33°–70° range; 2. AVT would be effective in treating multiple single and double AIS curve types in the thoracic and thoracolumbar/lumbar spine; 3. AVT would be effective across a broad range of skeletal immaturity, spanning the Risser skeletal maturity assessment, but the degree of correction achieved and the types of complications encountered would differ at the extremes of maturity; 4. The overall complication rate would be moderate in this initial series of AIS patients treated with AVT but certain complications would be reduced by a refinement of indications.

## Methods

Under IRB approved protocols, a retrospective analysis was performed on all AIS patients consecutively treated with AVT from 2010–2020 with 2 year follow-up. The overall range of curve magnitude was 33°–70° with skeletal maturity spanning Risser -1 to 5 (Risser -1 indicating Risser 0 with open triradiate cartilages) and Sanders 2–8. Both single and double curve patterns were treated in the thoracic and thoracolumbar/lumbar spine spanning Lenke types 1, 2, 3, 5, and 6. Charts and radiographs were reviewed to establish basic demographic data and identify complications and additional procedures. All patients had standard posterior-anterior and lateral full length scoliosis radiographs and bending films pre-op. The Cobb method was used to measure curve magnitude on pre-op, post-op and final radiographs [66]. Thoracic kyphosis and lumbar lordosis were also measured at similar time points using Scoliosis Research Society guidelines with normal ranges of 20°–45° and 40°–60°, respectively. Skeletal maturity was assessed using the Risser sign with an additional hand film to assess bone age as warranted [67, 68]. Rotation was assessed according to Nash-Moe [69].

Satisfactory treatment with AVT during the study period was defined as a final overall curve correction of at least 5° with no requirement or indication for fusion surgery. Because a relative improvement of 5° is quite modest, outcomes were also graded in absolute terms using final curve magnitude. Mild curves (≤ 25°) were categorized as excellent, moderate curves (26°–39°) as good, more severe curves (40°–49°) as fair, and the most severe curves (≥ 50°), or those requiring fusion, as poor. Because the moderate category was broad, it was further stratified into good (+) (26°–29°) and good (−) (36°–39°). Curve correction was defined as the initial reduction in curve magnitude from pre-op to post-op or the ultimate reduction from pre-op to final. Curve control was defined as the subsequent change in curve magnitude from post-op to final. Complications were defined as either approach or implant related and divided into intra-op, early post-op (<30 days post-operative), and late post-op (≥30 days) time periods.

The surgical technique for this procedure has been described previously by our group [43, 58]. In brief, all patients were placed in a lateral decubitus position for surgery. A 3-portal endoscopic approach with single lung ventilation was used for thoracic curves, allowing routine access T5 to T12 but, on occasion, access proximally to T4 and distally to L1 or even L2. A mini-open approach was used for thoracolumbar/lumbar curves with access possible T9 to L4.

The diaphragm was preserved in all cases. Segmental vessels were sacrificed prior to bicortical screw placement under fluoroscopic guidance. All levels were instrumented with a single PET cord spanning Cobb end vertebra to end vertebra. The PET cord was tensioned under fluoroscopic guidance to achieve as much correction of disc angulation as possible with the goal of achieving a neutral disc. Tensioning proceeded from proximal to distal with maximal tension applied to the majority of the curve, including the apex, with less tension at the ends. A temporary chest tube was used to assist with reinflation of the lung and evacuation of blood and fluid from the chest but was usually removed, under appropriate circumstances, at the end of the procedure. These circumstances included no evidence of pneumothorax, hemothorax, or fluid collection on intra-operative chest X-ray; no Pleur-evac air leak to suggest parenchymal lung injury; and no significant risk of post-operative bleed due to a bleeding disorder or other issue. Importantly, repeat clinical evaluations post-operatively with standard auscultation and serial chest X-rays assured the maintenance of clear lung fields with no evidence of pneumothorax, hemothorax, or other fluid collection at discharge. Appropriate spinal cord monitoring was used in all cases. Patients with double curve patterns had both curves treated under one anesthetic with the thoracic curve treated first. An access surgeon was utilized for all procedures.

Hydroxyapatite coated titanium screws and a polyethylene-terephthalate (PET) cord from the Dynesys Dynamic Stabilization System (Zimmer Biomet Spine, Broomfield, CO) were used in all cases without the polycarbonate-urethane spacer. As this device system was approved by the FDA in 2003 for adult lumbar spine stabilization as an adjunct to fusion, its use in the treatment of scoliosis in children during the study time period was considered an off-label indication.

Standard statistical analysis was performed using paired and one-sided t-tests, where appropriate, to determine the significance of pre-op versus post-op or final radiographic measurements. All statistical analyses were conducted in Microsoft Excel for Mac (version 16.52; Microsoft) with an alpha set at 0.05.

## Results

**Fig. 1:**
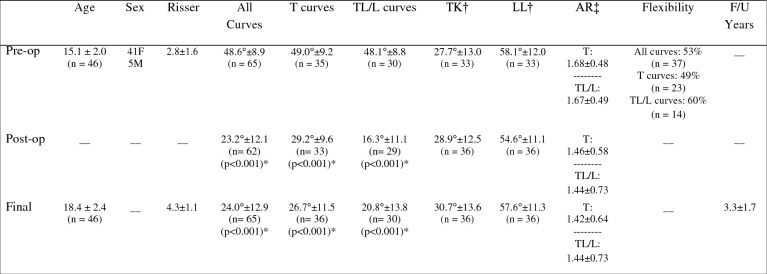
Demographic Data and Radiographic Measurements for the 46 AIS Patients Satisfactorily Treated with AVT. *Significant scoliosis correction was achieved pre-op to post-op and pre-op to final but the change in curve magnitude from post-op to final was not significant. †Thoracic kyphosis (TK) and lumbar lordosis (LL) remained within a normal range with no significant change pre-op to post-op to final. ‡Axial rotation (AR) according to Nash-Moe demonstrated a trend toward improvement in both T and TL/L curves but with no significant change pre-op to post-op to final

**Fig. 2:**
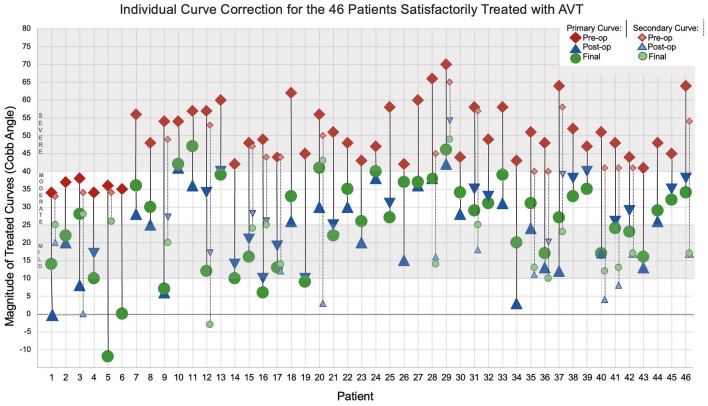
Individual curve correction for the 46 patients Satisfactory Treated with AVT. Of 52 AIS patients with 2-year follow-up after AVT, 46 were satisfactorily treated with initial curve correction (red diamond to blue triangle) and subsequent curve control (blue triangle to green circle) of the primary and secondary curves shown for each patient. The direction of the blue triangle (up or down) indicates the subsequent change in curve magnitude over time either with additional correction of the scoliosis with growth modulation (downward) or loss of correction (upward). Skeletal maturity using the Risser assessment is provided for each patient: R = − 1 (3, 6); R = 0 (4, 5, 37); R = 1 (1, 12, 13, 28, 33, 34, 36); R = 2 (2, 21, 32, 38, 39); R = 3 (20, 29, 41, 42); R = 4 (7, 9–11, 14–19, 22–27, 30, 31, 35, 40, 43–46); R = 5 (8). Significant curve flexibility was demonstrated on a bending film (≥ 50% or ≤ 30°) in all patients except 10, 12, 13, 20, 25, 27–29, 31, 38, 46. Tether rupture was demonstrated in patients 1, 7, 8, 11, 15, 16, 20, 26, 34, 35, 37, 40, 41

**Fig. 3:**
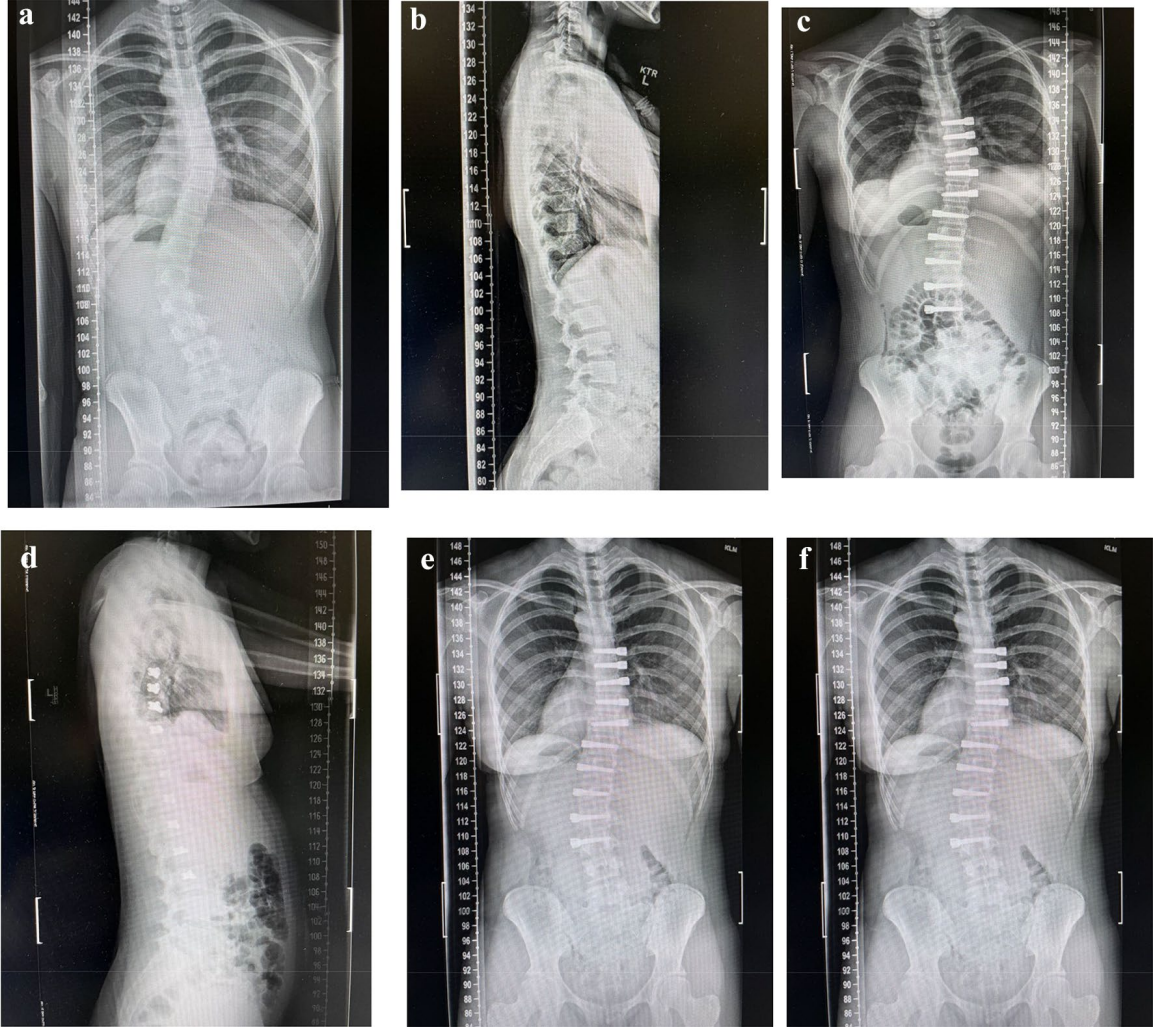
**A**–**F** (TIFF images 1–6). A 15 year old skeletally immature female with progressive 40° right thoracic and 48° left thoracolumbar curves (Lenke 6C) at Risser 1 was treated with AVT right T5-T10 and left T11-L3 with initial curve correction to 20° and 14°, respectively, and subsequent additional correction with growth over 2 years to 10° and 17°, respectively, at Risser 4. The post-operative left shoulder elevation resolved over time. [**A**–**B** (TIFF images 1–2) Pre-operative posteroanterior and lateral radiographs; **C**–**D** (TIFF images 3–4) Postoperative posteroanterior and lateral radiographs; **E**–**F** (TIFF images 5-6) Follow-up posteroanterior and lateral radiographs at 2 years]

Of 74 consecutive AIS patients treated with AVT, 52 patients (47 female, 5 male) with 74 curves had sufficient 2 year follow-up data for inclusion. Forty-six of these 52 patients (88%) with 65 curves (35T, 30TL/L) were satisfactorily treated with AVT, demonstrating an average overall curve correction from 48.6° pre-op (range 33-70°) at an age of 15.1 years (range 9.2–18.8) and skeletal maturity of Risser 2.8 (range Risser -1 to 5) to 23.2° post-op (range 0°–54°) and 24.0° final (range 0°–49°) at 3.3 years average follow-up (range 2–10 years) (Fig. 1). Of 46 patients satisfactorily treated with AVT at 2 year follow-up, 13/46 (28%) with 5 year follow-up demonstrated curve correction from 46.4° pre-op at an age of 15.0 years and Risser 2.5°–24.6° post-op to 29.1° final. Two of 46 (4%) with 8 year follow-up demonstrated curve correction from 34.7° at an age of 14.1 years and Risser 1.5 to 13.3° post-op to 20.3° final. Curve corrections from pre-op to post-op and pre-op to final were significant at 2 and 5 years (p<0.001) but did not reach significance at 8 years. The small changes from post-op to final at 2, 5, and 8 years were not significant but did represent reasonable control of scoliosis correction over time. A graphic representation of curve correction is provided for each individual patient (Fig. 2) as well as sample radiographs for patient 36 (Fig. 3). Thoracic kyphosis and lumbar lordosis were maintained in a normal range throughout the study with final measures of 31° and 58°, respectively. Axial rotation according to Nash-Moe demonstrated a slight trend toward improvement from 1.7 to 1.5. Skeletal maturity of Risser 4 or greater was achieved in all but one patient at study completion.

**Fig. 4:**
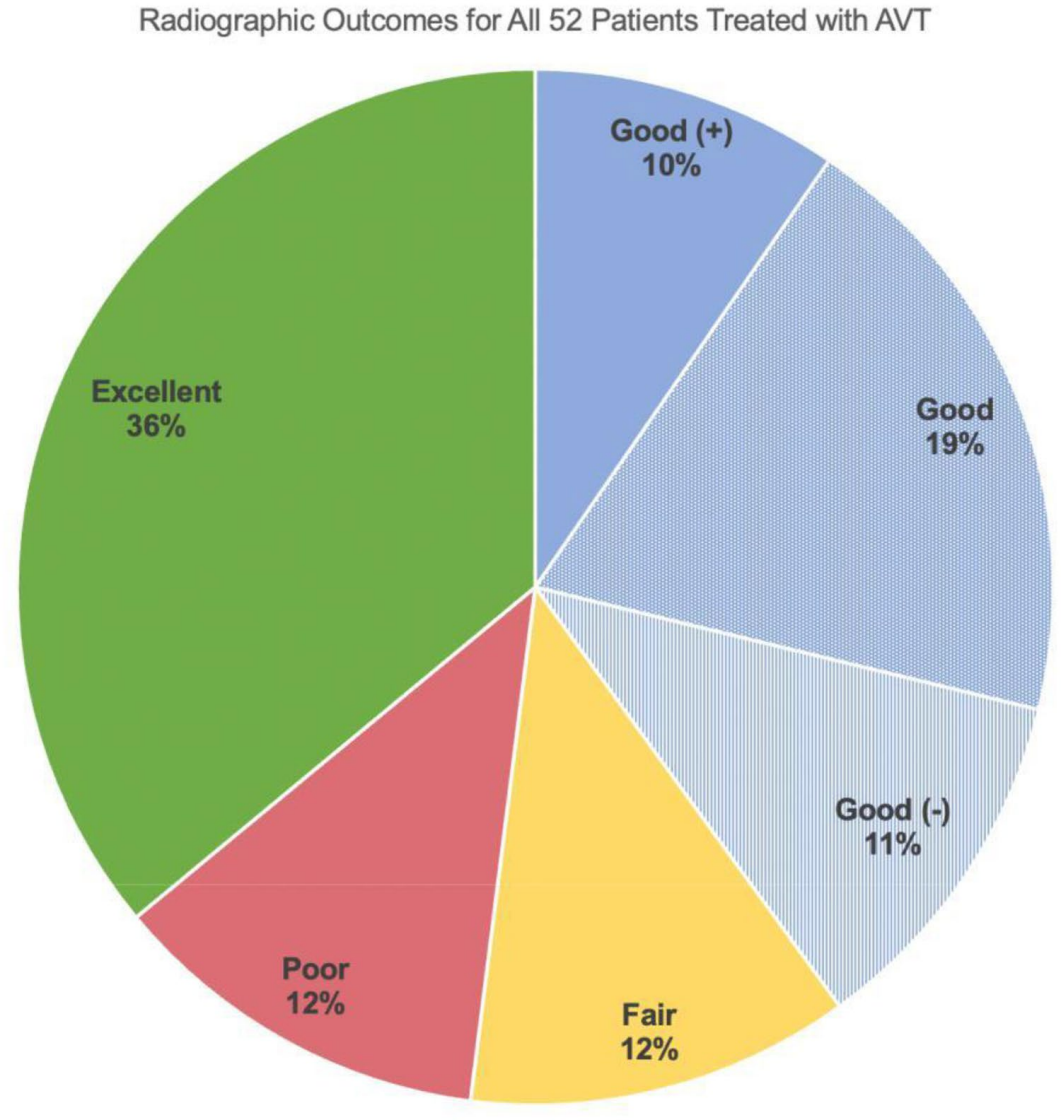
Radiographic outcomes were graded using final curve magnitude in all 52 patients with 74 curves treated with AVT: excellent (≤ 25°), good(+) (26–29°), good (30–35°), good (−) (36–39°), fair (40–49°), and poor (≥ 50°)

Radiographic outcomes were graded as excellent in 36% of patients (≤ 25° in 19/52), good in 40% (26°–39° in 21/52), fair in 12% (40°–49° in 6/52), and poor in 12% (≥ 50° or requiring fusion in 6/52) (Fig. 4). In those 40% of patients achieving a good outcome, 24% were graded as good (+) (26–29°) and 28% as good (−) (36–39°) with the remaining 48% in the 30–35° range.

Overall, of the 52 patients treated with AVT, 76% had final curves ≤39° and were graded as good or excellent, with 65% ≤35°, 48% ≤30°, and 36% ≤25°.

**Fig. 5:**
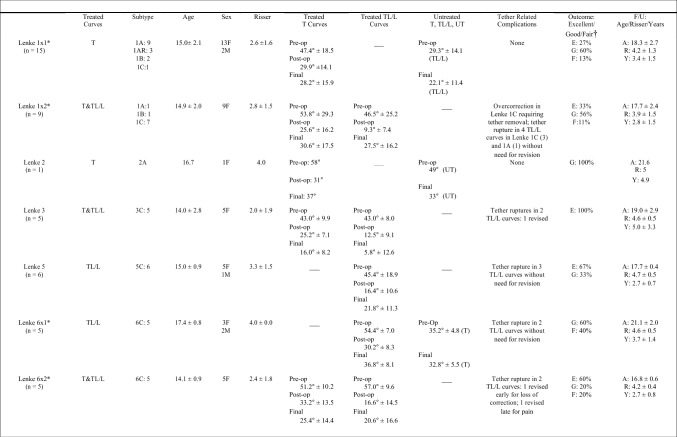
Outcomes by Curve Type, Number of Curves Treated, and Curve Location for the 46 AIS Patients Satisfactorily Treated with AVT. *Lenke 1 × 1 and Lenke 6 × 1 denote treatment of 1 curve only (T or TL/L); Lenke 1 × 2 and Lenke 6 × 2 denote treatment of two curves (T and TL/L). † Final curve magnitude was used to grade radiographic outcomes in 46/52 (88%) patients satisfactorily treated without fusion as excellent (≤ 25°), good (26°–39°), or fair (40°–49°); poor outcomes (≥ 50° or requiring fusion) in 6/52 (12%) patients not considered satisfactorily treated are included in Fig. 6

In the 46 satisfactorily treated patients with multiple single and double curve patterns, the distribution of curve types included Lenke 1 (24), Lenke 2 (1), Lenke 3 (5), Lenke 5 (6), and Lenke 6 (10) (Fig. 5). Though a single curve was consistently treated in all Lenke 2 and 5 patients, and both curves in all Lenke 3 patients, Lenke 1 and 6 patients had treatment of either one or both curves depending on curve magnitude. More specifically, 72% of Lenke 1 patients required treatment of the thoracic curve alone while 60% of Lenke 6 required treatment of both curves. All curve types demonstrated significant initial correction and subsequent control of scoliosis over time, but Lenke 3 curves demonstrated the best combined correction and control of deformity with the highest proportion of excellent radiographic results (100%).

Four of the 52 patients (8%) required additional procedures for tether rupture (3 replacements) or overcorrection (1 removal) to achieve satisfactory treatment status after AVT. An additional 6 of the 52 patients (12%), however, were not satisfactorily treated with AVT and required conversion to posterior spinal fusion for overcorrection (2) or inadequate correction (4).

**Fig. 6:**
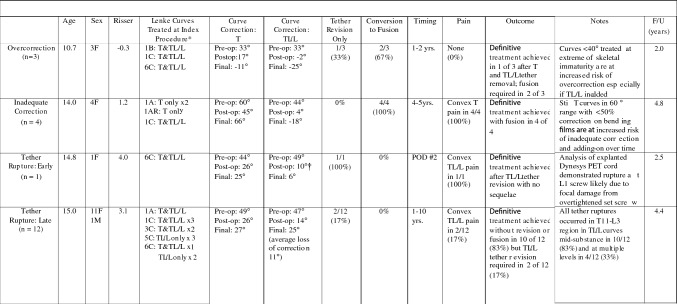
Tether Related Complications and Additional Procedures in All 52 AVT Patients with 2-Year Follow-Up. *Although the numbers were small, a relationship was noted between curve type and type of complication: all overcorrections were encountered in Lenke 1 and 6 double curve patterns in which both curves were small and flexible and both were treated; all inadequate corrections were encountered in Lenke 1 curves with a stiff T curve; all tether ruptures were encountered in TL/L curves and, thus, were more common in Lenke 5 and 6 curves; treatment of the TL/L curve alone in Lenke 5 and 6 was correlated with the highest tether rupture rate; Lenke 1 and 2 curves that only required treatment of the T curve demonstrated no tether ruptures. †Denotes corrected curve magnitude (10°) after immediate tether revision to address acute 32° loss of TL/L curve correction from 15° to 47° T12-L2 due to tether rupture at L1 screw on POD#2

**Fig. 7:**
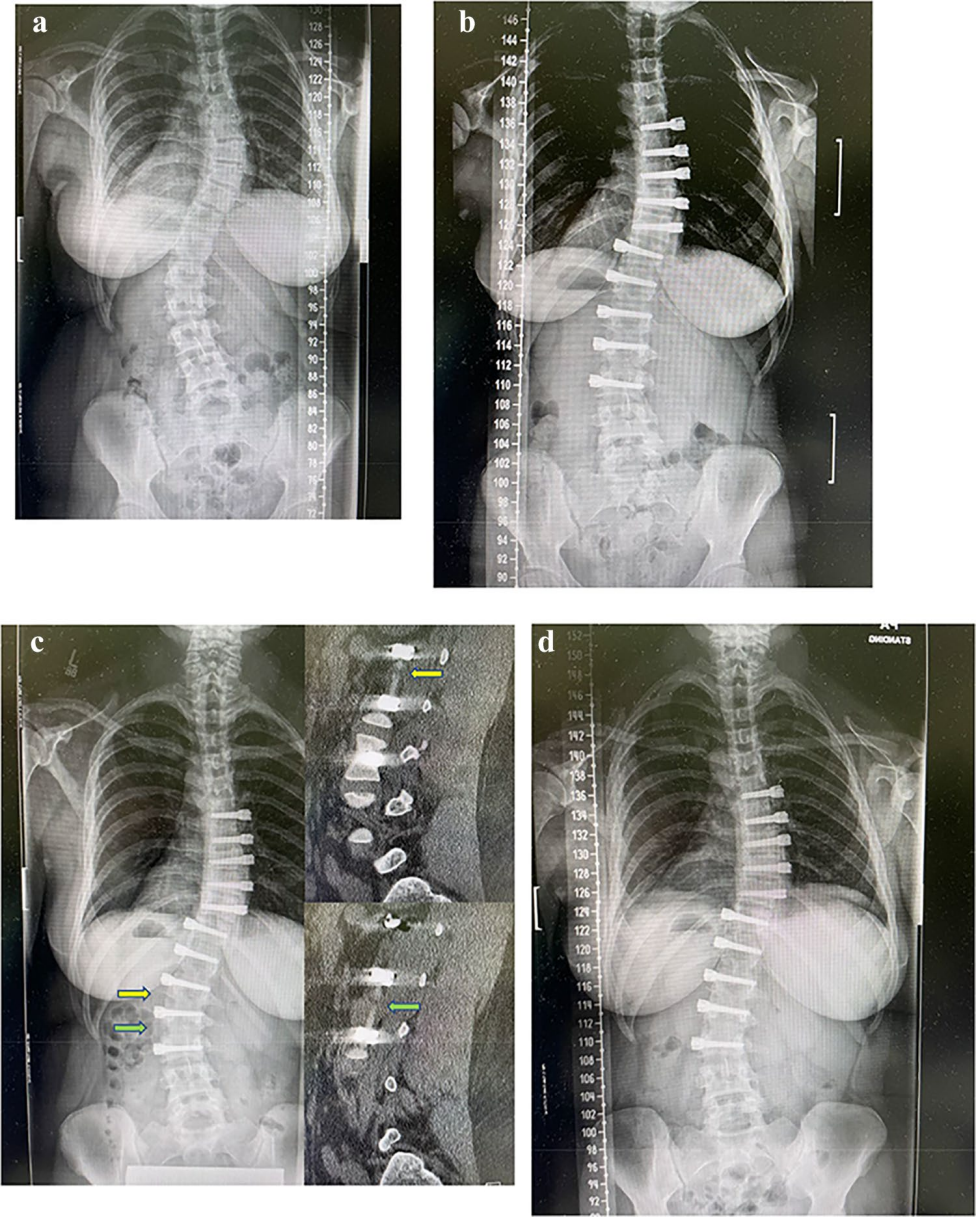
**A**–**D** (TIFF images 6–10). A 14 year old relatively skeletally mature female with progressive 47° right thoracic and 48° left thoracolumbar curves (Lenke 3C) at Risser 4 was treated with AVT right T6-10 and left T11-L3 with initial curve correction to 28° and 21°, respectively, with good control of both curves over 2 years. At 2 years, the thoracolumbar curve demonstrated 7° loss of correction to 28° with little change in the thoracic curve. Thoracolumbar tether rupture was suspected due to the change in angulation within screw pairs at L1,2 and L2,3. Mid-substance tether ruptures were confirmed at both levels on sagittal CT reconstructions. Despite a lack of pain or other symptoms, the patient and family were interested in revision AVT surgery. The tether was replaced uneventfully T11-L3 with curve correction to 24° and 16°, respectively. [**A** (TIFF image 7) Pre-operative posteroanterior radiograph; **B** (TIFF image 8) Postoperative posteroanterior radiograph; **C** (TIFF image 9) Follow-up posteroanterior radiograph at 2 years suggesting tether ruptures L1-3 and two CT sagittal reconstructions confirming tether ruptures at L1,2 and L2,3; **D** (TIFF image 10) Follow- up posteroanterior radiograph 1.5 years after tether revision]

The overall implant related complication rate in all 52 patients was 38% with 8% requiring tether revision and 12% requiring conversion to fusion (Fig. 6). The remaining 18% represented stable, asymptomatic tether ruptures that did not require treatment. The distribution of implant related complications over time revealed 0% (0/52) intraoperative complications; 2% (1/52) early complication with a single immediate tether rupture; and 36% (19/52) late post-operative complication (12 late tether ruptures, 3 overcorrections, 4 inadequate corrections). Overall, the 38% (20/52) implant related complications included 6% (3/52) overcorrections, 8% (4/52) inadequate corrections, and 25% (13/52) tether ruptures. Though tether ruptures were evident in 25% of patients with 2 year follow-up (13/52), 54% of patients with 5 year follow-up (7/13) and 50% of patients with 8 year follow-up (1/2) demonstrated evidence of tether rupture. Problematic tether rupture requiring tether revision occurred in 2/52 (4%) at 2 years, 1/13 (8%) at 5 years, and 0/2 (0%) at ten years. While all 3 overcorrections required additional surgery (1 tether removal alone; 1 tether removal followed by remote posterior spinal fusion; and 1 posterior spinal fusion without tether removal), and all 4 patients with inadequate correction required conversion to fusion, only 3 of 13 tether ruptures required revision (1 revision for immediate thoracolumbar/lumbar tether rupture on POD#2 resulting in significant, symptomatic loss of correction; and 2 revisions for late thoracolumbar/lumbar tether rupture at 2 and 5 years, neither with significant loss of correction but one with convex lumbar pain). An example of tether revision for asymptomatic tether rupture is shown in patient 15 (Fig. 7). The approach related complication rate was 4% (2/52) with 2 pleural effusions at 1–2 weeks post-op that both resolved with percutaneous drainage.

## Discussion

In this retrospective study of our first 52 patients treated over a 10 year period, 88% of AIS patients were considered satisfactorily treated with AVT at 2–10 years as they not only avoided fusion surgery but fusion was not indicated or contemplated. Further, the average curve correction of 24.6° resulted in significant correction of the deformity from 48.6° pre-op to 24.0° final (p < 0.001) with all satisfactorily treated patients achieving the minimum threshold of at least 5° correction from pre-op to final curve magnitude. Importantly, however, only 68% of 52 patients achieved a good or excellent result after a single surgical intervention, as 8% required an additional procedure (tether replacement or removal) to achieve satisfactory status and 12% had final curves in the fair range (40°–49°). Though this 12% only achieved a fair outcome, the average final curve correction of 13° in this group reduced curve magnitude from 57° to 44° allowing the avoidance of fusion surgery to date.

Unfortunately, an additional 12% of patients were not satisfactorily treated with AVT as fusion surgery was required for overcorrection in 4% and inadequate correction in 8%. These results were not inconsistent with other published studies. Samdani [57] reported the highest rate of satisfactory treatment in 98% of patients at 4–5 years with a revision surgery rate of 12% but only 2% requiring fusion. Newton [51] reported satisfactory treatment in 59% of AIS and syndromic patients at 2–4 years with overcorrection in 24% and fusion in 18%. While the rates of complications and additional procedures differ significantly in these studies, this can be partially explained by differences in curve magnitude, curve type, and skeletal immaturity, with Newton’s study having larger curves, greater immaturity, and more variation in curve type and underlying diagnosis.

While success in our study was defined broadly as the satisfactory treatment of scoliosis without progression or the need for fusion, the overall 50% relative correction in curve magnitude from 48.6° pre-op to 24.0° at final follow-up was significant (p < 0.001) and consistent with the corrections reported by Samdani [57] and Newton [51], at 54% and 51%, respectively, but less than Hoernschemeyer [56] at 82%. The absolute final correction by Samdani to < 30° in 80% of patients was the highest reported, with Hoernschemeyer reporting 74% ≤ 30° and Newton reporting 59% ≤ 35°. Again, these differences are partially explained by the larger curves treated by Newton but may also be related to a greater degree of skeletal immaturity and a higher rate of complications. Though our curve corrections revealed 76% ≤ 39°, 65% ≤ 35°, 48% ≤ 30° and 36% ≤ 25°, we treated a broader range of curve types with a number of larger and, potentially, stiffer curves than most studies with less chance of growth modulation over time due to greater skeletal maturity.

This study also demonstrated reasonable correction and control of scoliosis in multiple Lenke curve types and represents the only study to our knowledge with 2–10 year follow-up on multiple double curve patterns (Lenke 1B, 1C, 2, 3, and 6). The best correction and control in this study was demonstrated in Lenke 3 curves with 100% achieving an excellent (≤ 25°) radiographic outcome despite 2 thoracolumbar/lumbar tether ruptures. No other significant complications were encountered in this group. While the exact reason for the high rate of success in Lenke 3 curves is not clear, it may be due, in part, to the fact that all of these patients met what we consider to be ideal indications for tether surgery: curves in the 40–60° range, ≥ 50% flexibility, and skeletal maturity in the Risser 0–2 (Sanders 3–5) range [70]. These factors likely allowed for significant initial correction of both thoracic and thoracolumbar/lumbar deformities with subsequent additional control of these curves with growth modulation over time. Controlling progression in both curves in each patient may have been an additional factor contributing to the success in this group.

Although 88% of patients in this study were considered satisfactorily treated with AVT, this group was not free of complications or additional procedures. The most common complication, tether rupture, occurred in 13 (25%) patients but required revision surgery in only 3 (6%) with no tether rupture patient requiring conversion to fusion. The 10 patients with asymptomatic tether rupture and 11 degrees average loss of correction (5°–40°) remained stable radiographically (no evidence of progressive loss of correction over time) and clinically (fully functional without limitations) during the study period without any current indication for revision surgery. Though Samdani [57] recently reported no relevant tether ruptures in 57 patients at 4–5 years, Newton [51] and Hoernschemeyer [56] have reported tether rupture rates as high as 47–48%. Newton’s tether revision rate, however, was only 6%, but conversion to fusion for tether rupture was required in up to 24%. Hoernschemeyer’s tether revision rate was 21% with only 7% requiring conversion fusion. Though our tether rupture rate falls somewhere in the middle of these other studies, a direct comparison is difficult as curve types and tether rupture locations were different in our study. Further, the need for conversion to fusion was not required in our tether rupture patients likely because they were somewhat more mature initially and often encountered tether rupture after the achievement of skeletal maturity. Additionally, while most other studies have primarily treated thoracic curves and, thus, noted only thoracic tether ruptures, this study, in which multiple single and double curve patterns were treated, revealed a significantly different location for tether rupture. All tether ruptures in this study occurred in the thoracolumbar/lumbar region (T11-L3) in thoracolumbar/lumbar curves with no ruptures in the thoracic curves.

In contrast to tether rupture, the phenomenon of overcorrection requiring revision surgery was confined to patients with smaller curves (<40°) in the setting of significant skeletal immaturity (often Risser -1). All 3 significant overcorrections were encountered in our first 10 patients who had an average age of 10.7 years at Risser –0.3 at the time of treatment. These 3 patients corrected from 33° pre-op to 7.5° post-op to -18° final at 2 years. These two factors, combined with substantial initial correction of flexible curves, were likely responsible for all 3 overcorrections. Though overcorrection required revision surgery in all three patients, only 2 required fusion while the third stabilized with curves <30° after tether removal.

Inadequate correction requiring conversion to fusion was encountered in 8% (4/52) of patients with large thoracic curves and limited flexibility. In these patients, pre-op bending films demonstrated a decreased average flexibility of 36%, resulting in suboptimal average correction of these curves from 60.5° pre-op to 45.3° post-op to 61.3° final. The subsequent progression of deformity in these patients, however, was not due to tether rupture but, rather, caused by adding on above and below an intact tether.

A final note regarding the treatment of more mature AIS patients with AVT seems appropriate given the inclusion of many of these patients in this study. Though controversial, the treatment of more mature adolescents is not only logical and sound, but is based upon standard principles that guide all surgical treatments for scoliosis. That is, to achieve significant correction and control of the deformity with minimal negative impact on the normal physiology and biomechanics of the spine. AVT offers the possibility of achieving these goals even in the absence of significant remaining growth. Although targeting growth modulation is attractive in the treatment of more immature AIS patients, as it can substantially augment the initial scoliosis correction achieved with AVT at the time of surgery, it is not the goal of AVT. Growth modulation is merely one of the tools that can be exploited to help achieve the goals of deformity correction and control.

There are, essentially, three arguments that support the treatment of more mature AIS patients with AVT. First, the primary goal of AVT is to treat scoliosis without fusion. While ideal candidates for AVT (curves 40°–60°, ≥ 50% flexibility, and Risser 0–2) appear to have the highest chance of success, acceptable candidates (curves 40°–60° and ≥ 50% flexibility but Risser > 2) can still achieve significant correction and control of scoliosis without fusion and, thus, without sacrificing the motion, function, and overall health of the spine [70]. In our experience, the acceptable candidates for AVT will have a slightly larger final curve magnitude than the ideal candidates, probably related to a higher incidence of tether rupture, but with a reduced risk of revision surgery compared to more immature patients. Though the potential benefits of AVT in more mature patients can be seen after treatment of thoracic and thoracolumbar/lumbar curves, the most dramatic differences between AVT and fusion are evident after treatment of curves that involve the lumbar spine. Not only does AVT potentially preserve significant lumbar motion and function, but maintained flexibility in the lumbar region after AVT likely reduces the risk of adjacent segment degeneration and related revision surgeries. Second, though it is important to acknowledge that growth modulation can contribute to success after AVT, it may not be an essential ingredient. As most studies to date have demonstrated that approximately 80% of the final scoliosis correction is achieved at the time of surgery, the 20% contribution from growth modulation may not be necessary in all patients to achieve overall success. Third, though growth modulation is often a visible phenomenon that can be measured on serial radiographs, it is probably not the only type of modulation that occurs after AVT. More subtle types of modulation in the osseous tissues, according to Wolff’s Law [71, 72], or even in the soft tissues, according to Davis’ Law [73, 74], may not be visible, but certainly could impart some degree of internal stability, via remodeling along the lines of force, to the corrected scoliosis over time. Though these more subtle types of modulation have not been extensively studied in scoliosis, attempts have been made to better understand their significance [75]. These secondary forms of tissue modulation have implications for the maintenance of curve correction over time even after tether rupture in these more mature AIS patients.

The limitations of this study are not insignificant. First, because this study represents the earliest series of patients ever treated with this novel fusionless scoliosis surgery, the indications, by necessity, evolved over the 10-year study period. This occurred primarily due to the success of the tether in correcting, and even overcorrecting, scoliosis in smaller curves (< 40°) at the extreme of skeletal immaturity (Risser -1). Our subsequent success in treating curves in the 40°–60° range at Risser 0–2, without overcorrection, eventually led to consideration of tethering for some flexible curves > 60°. Additionally, the significant initial correction achieved in most curves at the time of surgery, especially in the thoracolumbar/lumbar region, allowed for the treatment of more mature adolescents interested in avoiding an extensive lumbar fusion. Second, given the FDA restrictions on prospective analysis of patients undergoing treatment with devices used off-label, retrospective analysis was the most reasonable study method available to our group for review of the entire 10 year experience. Third, the retrospective nature of this study may have adversely affected the percentage of 2 year follow-up patients, which was less than optimal at 70%. Fourth, the study lacked a patient reported outcomes instrument. Despite these limitations, however, this study provides valuable insight into a novel, fusionless surgical treatment that achieves satisfactory correction of scoliosis in the majority of patients.

Additional study will be necessary to further improve the safety and efficacy of this new procedure. FDA HDE approval of this tether device in 2019 has already facilitated a higher level of analysis involving prospective studies with well-matched controls and robust outcomes instruments.
